# Sex differences in predicting ADHD clinical diagnosis and pharmacological treatment

**DOI:** 10.1007/s00787-018-1211-3

**Published:** 2018-08-10

**Authors:** Florence D. Mowlem, Mina A. Rosenqvist, Joanna Martin, Paul Lichtenstein, Philip Asherson, Henrik Larsson

**Affiliations:** 1 Social, Genetic, and Developmental Psychiatry Centre (SGDP), Institute of Psychiatry, Psychology and Neuroscience, King’s College London, DeCrespigny Park, Denmark Hill, London, SE5 8AF UK; 2 Department of Medical Epidemiology and Biostatistics, Karolinska Institutet, Stockholm, Sweden; 3 MRC Centre for Neuropsychiatric Genetics and Genomics, Cardiff University, Cardiff, UK; 4 School of Medical Sciences, Örebro University, Örebro, Sweden

**Keywords:** Attention-deficit/hyperactivity disorder/ADHD, Sex differences, Clinical diagnosis, Population-based study

## Abstract

In youth, ADHD is more commonly diagnosed in males than females, but higher male-to-female ratios are found in clinical versus population-based samples, suggesting a sex bias in the process of receiving a clinical diagnosis of ADHD. This study investigated sex differences in the severity and presentation of ADHD symptoms, conduct problems, and learning problems in males and females with and without clinically diagnosed ADHD. We then investigated whether the predictive associations of these symptom domains on being diagnosed and treated for ADHD differed in males and females. Parents of 19,804 twins (50.64% male) from the Swedish population completed dimensional assessments of ADHD symptoms and co-occurring traits (conduct and learning problems) when children were aged 9 years. Children from this population sample were linked to Patient Register data on clinical ADHD diagnosis and medication prescriptions. At the population level, males had higher scores for all symptom domains (inattention, hyperactivity/impulsivity, conduct, and learning problems) compared to females, but similar severity was seen in clinically diagnosed males and females. Symptom severity for all domains increased the likelihood of receiving an ADHD diagnosis in both males and females. Prediction analyses revealed significant sex-by-symptom interactions on diagnostic and treatment status for hyperactivity/impulsivity and conduct problems. In females, these behaviours were stronger predictors of clinical diagnosis (hyperactivity/impulsivity: OR 1.08, 95% CI 1.01, 1.15; conduct: OR 1.43, 95% CI 1.09, 1.87), and prescription of pharmacological treatment (hyperactivity/impulsivity: OR 1.24, 95% CI 1.02, 1.50; conduct: OR 2.20, 95% CI 1.05, 4.63). Females with ADHD may be more easily missed in the ADHD diagnostic process and less likely to be prescribed medication unless they have prominent externalising problems.

**Electronic supplementary material:**

The online version of this article (10.1007/s00787-018-1211-3) contains supplementary material, which is available to authorized users.

## Introduction

Attention-deficit/hyperactivity disorder (ADHD) is a neurodevelopmental disorder characterised by age-inappropriate and maladaptive levels of inattention and/or hyperactivity/impulsivity. In children and adolescents, ADHD is more commonly diagnosed in males, with the sex ratio ranging from 2:1 to 10:1 [1–5]. However, sex ratios appear to be dependent on the type of sample, with higher male-to-female ratios found in clinical versus population-based samples. Furthermore, the male-to-female ratio is smaller in adult clinic samples than in childhood and adolescent samples [6]. This suggests that, in youth, ADHD affects a greater proportion of females than reflected in clinical practice and that differences exist in the diagnostic process for males and females with ADHD symptoms [7, 8].

The findings relating to sex differences in ADHD are variable and sometimes contradictory, partly due to differences in sample characteristics. Meta-analyses tend to show less severe symptoms in females versus males with ADHD identified from non-referred, community populations, but similar levels in clinically ascertained samples—with the exception of inattention for which females had higher ratings in the more recent meta-analysis [5, 9]. Several other studies have also not found support for sex differences for ADHD symptoms and co-occurring problems in clinical and referred samples [1, 10]. While important information has been gained from both population-based and clinical samples of children with ADHD, the approach of investigating sex differences in either a population-based or clinical sample means that it is not clear what factors are specifically leading to the clinical diagnosis of children with ADHD from the population, and if these differ as a function of sex.

Sex differences in the phenotypic expression of ADHD are often proposed as an explanation for the greater rates of ADHD diagnosis in males. A common hypothesis is that females with ADHD are more likely to present with predominantly inattentive symptoms, and less hyperactive/impulsive or conduct problems than boys, and are thus perceived as less problematic [4, 7, 11]. Therefore, females with ADHD problems that manifest as predominantly inattentive symptoms and lower levels of disruptive behaviours may be less likely to receive a diagnosis of ADHD [5].

Studies also show sex differences in the pattern of ADHD treatment, with males being more likely to receive ADHD medication than females [12, 13]. However, the underlying reasons for the observed sex differences in treatment remain to be investigated. Different pharmacological treatment rates in males and females could also be due to a different manifestation of the disorder. It is important to understand whether certain symptom manifestations have greater influence on being prescribed pharmacological treatment, and the possibility that females with ADHD are undertreated is an important public health concern [8].

Another consideration in the diagnostic and treatment process of individuals with ADHD is the presence of co-occurring learning problems, since learning problems represent another leading reason for identification of children with ADHD. Research has demonstrated that females with ADHD are less likely to have learning difficulties or manifest problems at school compared to males [14, 15], which could also lead to lower identification of ADHD in females. Sex differences in learning problems related to ADHD, and their impact on the diagnostic and treatment process, are not well investigated.

This study investigated sex differences in ADHD using a large population-based sample [The Child and Adolescent Twin Study in Sweden (CATSS)] linked to Swedish National Patient Register data on clinical ADHD diagnoses and prescribed ADHD medications. Thus, enabling investigation of a population-based and clinical sample for which there is not an ascertainment bias and overcoming important limitations of studies reliant on one type of sample alone. We first described the severity of ADHD symptoms, conduct, and learning problems in males and females with and without clinically diagnosed ADHD, followed by examination of the ADHD symptom presentation. We then investigated whether the predictive associations of inattention, hyperactivity/impulsivity, conduct problems, and learning problems on being diagnosed and treated for ADHD differed in males and females. It was hypothesised that (1) at the population level, males would show greater symptom severity than females, but at the clinical level similar severity would be observed, with the exception of inattention for which levels may be higher in females as suggested by meta-analysis, (2) hyperactivity/impulsivity and conduct problems would be a stronger predictor of diagnosis in females than in males and inattention a weaker predictor, and (3) in children with a clinical diagnosis of ADHD, hyperactivity/impulsivity and conduct problems would be a stronger predictor of medication status in females than males.

It is important to increase our understanding of sex effects in ADHD and whether certain symptoms are more predictive of clinical diagnosis and pharmacological treatment (including whether sex differences in such predictors exist), as it can lead to improved identification of females with the disorder. Furthermore, it may point towards certain biases in the diagnostic and treatment process which has implications for clinical practice and can inform our understanding of the way that clinicians recognise ADHD symptoms, and potentially apply the diagnostic criteria.

## Methods

### Sample

Participants were from The Child and Adolescent Twin Study in Sweden (CATSS) [16], an ongoing prospective longitudinal cohort twin study that targets all twins in Sweden born since 1992. A telephone interview is conducted with parents of all twins, no more than 1 month before or after their 9th or 12th birthdays (CATSS-9/12; baseline). For the present study, data from 19,804 CATSS children assessed at age 9 years were available for analyses (50.64% males). The CATSS-9/12 study has ethical approval from the Karolinska Institute Ethical Review Board and participants are protected by the informed consent process.

### Measures

*ADHD symptoms and co*-*occurring behavioural traits* The Autism-Tics, AD/HD and other Comorbidities Inventory (A-TAC) was administered to parents of twins over the telephone, and questions were asked from a lifetime perspective. The A-TAC is a broad screening instrument that encompasses multiple neurodevelopmental disorders. Two modules of the A-TAC are used to assess ADHD (one assessing inattention [9 items] and one assessing hyperactivity/impulsivity [10 items]), consisting of a total of 19 items that correspond closely to DSM-5 diagnostic criteria for ADHD [17]. Questions are answered on a 3-point scale: ‘no’ (scored as 0), ‘yes, to some extent’ (scored as 0.5), and ‘yes’ (scored as 1). Thus, the maximum score that can be obtained is 19. These questions were identified to achieve the optimal sensitivity, specificity, and predictive value for clinical ADHD diagnoses in validation studies, with high internal consistency [18–21].

Using the A-TAC ADHD items, it is possible to categorise individuals based on DSM-5 symptom criteria for the three ADHD presentations: the predominately inattentive presentation, based on the presence of six or more symptoms of inattention; the predominantly hyperactive/impulsive presentation, based on the presence of six or more symptoms of hyperactivity/impulsivity (using nine of the ten A-TAC items); and the combined presentation, based on six or more symptoms of both inattention and hyperactivity/impulsivity. From the three-point scale described above, we dichotomised responses for each item into ‘symptom present’ (‘yes’ and ‘yes to some extent’ were collapsed into one category) and ‘symptom absent’ to enable categorisation of participants into one of the three ADHD presentations.

The A-TAC also includes questions that target other well-described clinical features of psychiatric disorders, such as conduct problems (five items relating to lying, cheating, stealing, being cruel, or starting fights) and learning difficulties (three items relating to reading and maths skills and slow learning), also scored on a three-point scale as above. Thus, whilst looking specifically at one disorder, co-occurring problems can also be examined. Of note, although the A-TAC also includes questions on anxiety and mood, we were unable to examine these variables due to a reduced number of CATSS participants completing these questions.

*Socio*-*Economic Status (SES)* Maternal education from the Swedish Register of Education was used as an indicator of socio-economic status. A categorical variable was created (low = primary and secondary education, ≤ 9 years; medium = upper secondary education, 10–12 years; high = post-secondary education, > 12 years).

### Population-based registers

Unique personal identifier numbers enable data from participants in the CATSS sample to be accurately linked with information from National population-based registers up until December 2013. Thus, it was possible to determine whether participants in CATSS had been referred to a specialist clinic and diagnosed with ADHD, and if they were prescribed ADHD medication. Registry data were also used to identify 273 participants in CATSS who had emigrated (obtained from The Migration Register) or died (obtained from The Cause of Death Register) after their participation in the study; these individuals were excluded from analyses.

*The National Patient Register (NPR)* The NPR contains information about all psychiatric inpatient (from 1987) and outpatient (from 2001) care in Sweden, from both private and public caregivers (primary care is not currently included). Clinical ADHD diagnoses are based on the International Classification of Diseases (ICD), code F90 [22], but most clinicians base their clinical assessment on DSM criteria for ADHD and recode to ICD. Participants were identified as having a diagnosis of ADHD from the NPR if they had at least one record of inpatient or outpatient care for ADHD from 2001 to 2013.

*Prescribed Drug Register (PDR)* The PDR contains data for all dispensed drug prescriptions to the entire Swedish population since July 2005. Information on the indication for the prescription is not recorded; however, ADHD is a group that can be identified as treatment is characterised by a few drugs exclusively used for this disorder (methylphenidate hydrochloride, atomoxetine, amphetamine sulfate, or dextroamphetamine sulfate). Participants treated with ADHD medication were identified if they had at least one prescription from 2005 to 2013.

### Statistical analysis

Descriptive statistics are presented to describe the severity and presentation of ADHD symptoms and co-occurring behaviours in males and females with and without clinically diagnosed ADHD. Sex differences in parent-rated ADHD symptom scores (inattention and hyperactivity/impulsivity) and co-occurring conduct and learning problems scores were tested using linear regression models (hypotheses 1), and sex differences in ADHD medication status was tested using logistic regression.

To assess whether the predictive associations of inattention, hyperactivity/impulsivity, conduct problems, and learning problems with clinical diagnoses differed in males and females, we used a series of logistic regression models (hypotheses 2). The models were conducted separately for each symptom domain (inattention, hyperactivity/impulsivity, conduct problems, and learning problems) using the continuous score, and stratified by sex. We also applied logistic regression models with males and females included in one model to investigate sex-by-symptom interactions on diagnostic status (again, separate models were run for each symptom domain using the continuous score).

Next, we used a series of logistic regression models to examine sex differences in the associations between these symptom domains and ADHD medication status in children with clinically diagnosed ADHD (hypotheses 3). The models were conducted separately for each symptom domain using the continuous score and stratified by sex. We also investigated sex-by-symptom interactions on treatment status.

All regression models were adjusted for the effects of year of birth and family SES. Furthermore, as the data were used as population data and not analysed in a twin analysis model, we controlled for the clustered data structure (to correct for the inclusion of two study children in each family) using a cluster-robust sandwich estimator (the *cluster(vce)* command in Stata [23]).

## Results

### Prevalence

In the CATSS sample, 3.28% (*n* = 650) of the children had a clinical diagnosis of ADHD recorded in the National Patient Register (NPR). Clinically diagnosed ADHD was more common in males (4.65%, *n *= 466) than in females (1.88%, *n *= 184), which corresponds to a prevalence ratio of ~ 2.5:1.

Based on DSM-5 ADHD symptom criteria using the parent-reported A-TAC questionnaire, 2556 individuals (12.9%) from the CATSS sample met criteria for ADHD. More males (16.3%, *n *= 1635) than females (9.43%, *n *= 921) met the symptom criteria, corresponding to a prevalence ratio of ~ 1.8:1. Among these children, 303 (18.5%) of the males with elevated symptoms, and 111 (12.1%) of the females with elevated symptoms had an ADHD diagnosis recorded in the NPR.

### Symptom severity

Table 1 shows mean symptom scores for children with and without a clinical diagnosis of ADHD in the NPR. Among non-diagnosed children, females had significantly lower scores compared to males for total ADHD, inattention, hyperactivity/impulsivity, conduct problems, and learning problems (*p* values < 0.001). In contrast, among children with clinically diagnosed ADHD, males and females showed similar severity across the symptom domains, except for significantly higher inattention scores in males (*p *= 0.03, *d *= 0.21). Females and males with a clinical diagnosis were equally likely to be prescribed ADHD medication.

**Table 1:** Characteristics of the non-diagnosed and clinically diagnosed children (means and SD unless otherwise stated)

Characteristic^a^	Non-diagnosed^b^					Diagnosed in the NPR				
	Overall (*n* = 19,154)	Males (*n* = 9563)	Females (*n* = 9591)	*P*	Cohen’s *d*	Overall (*n* = 650)	Males (*n* = 466)	Females (*n* = 184)	*P*	Cohen’s *d*
Total ADHD	1.85 (2.77)	**2.19 (3.02)**	**1.50 (2.45)**	**<.001**	0.25	8.44 (5.53)	8.72 (5.46)	7.75 (5.64)	.08	0.18
Inattention	0.94 (1.58)	**1.13 (1.72)**	**0.74 (1.41)**	**<.001**	0.25	4.43 (2.86)	**4.60 (2.80)**	**4.01 (2.97)**	**.03**	0.21
Hyperactivity/impulsivity	0.91 (1.53)	**1.06 (1.67)**	**0.76 (1.37)**	**<.001**	0.20	4.02 (3.17)	4.12 (3.17)	3.76 (3.18)	.25	0.11
Conduct	0.08 (0.31)	**0.10 (0.35)**	**0.07 (0.27)**	**<.001**	0.10	0.60 (0.99)	0.58 (0.97)	0.63 (1.03)	.50	− 0.05
Learning	0.25 (0.57)	**0.27 (0.59)**	**0.23 (0.55)**	**<.001**	0.07	1.01 (1.05)	0.98 (1.02)	1.11 (1.13)	.17	− 0.12
Medication % (*n*)	–	–	–	–	–	552	84.98% (396)	84.78% (156)	.93	–

Bold data signify statistical significance of the tests

All models were adjusted for familial clustering, year of birth, and SES

*ADHD* attention-deficit/hyperactivity disorder, *NPR* National Patient Register

^a^Data were missing on some variables; all available data were used in analysis

^b^Scores for the entire population can be found in the Supplementary Table 1

### ADHD presentations

Among all children from the CATSS sample meeting DSM-5 symptom criteria for ADHD as identified with the parent-reported A-TAC questionnaire, the inattentive presentation was most common (53.7%), followed by the combined (26.8%) and hyperactive/impulsive (19.5%) presentations (Table 2). A significantly greater percentage of females met symptom criteria for the inattentive presentation category compared to males (*χ*^2^(1) = 11.27, *p *= 0.002, *d *= 0.11), and a significantly greater percentage of males met symptom criteria for the combined presentation category compared to females (*χ*^2^(1) = 17.39, *p *< 0.001, *d *= 0.15). There was a similar percentage of males and females meeting symptom criteria for the hyperactive/impulsive presentation (*χ*^2^(1) = 0.19, *p *= 1.0, *d *= 0.08).

**Table 2:** ADHD presentations based on DSM-5 criteria symptom counts measured with parent-rated A-TAC, in the entire CATSS population and diagnosed children

Presentation; *n* (%)	Entire population^a^						Diagnosed in the NPR^b^					
	Overall	Males	Females	χ^2 c^	*P*	Cohen’s *d*	Overall	Males	Females	χ^2 c^	*P*	Cohen’s *d*
Inattentive	1372 (53.7)	837 (51.2)	535 (58.1)	11.27	**.002**	0.11	151 (36.7)	107 (35.6)	44 (39.6)	0.56	1.0	− 0.02
Hyperactive/impulsive	499 (19.5)	315 (19.3)	184 (20.0)	0.19	1.0	0.08	33 (8.0)	25 (8.3)	8 (7.2)	0.13	1.0	0.05
Combined	685 (26.8)	483 (29.5)	202 (21.9)	17.39	**<.001**	0.15	228 (55.3)	169 (56.1)	59 (53.2)	0.29	1.0	0.09

Bold data signify statistical significance of the tests

*NPR* National Patient Register

^a^Inclusive of children with a clinical diagnosis

^b^236 children (163 males) did not meet symptom criteria for any of the presentations based on the A-TAC

^c^Chi square tests are adjusted for multiple testing using Bonferroni correction

Looking exclusively among the children clinically diagnosed with ADHD in the NPR, the combined presentation was most common (55.3%), followed by the inattentive (36.7%) and hyperactive/impulsive (8.0%) presentations (Table 2). Among these cases, there were no statistically significant differences between males and females in the ADHD presentations (Table 2).

### Does the predictive value of ADHD symptoms, conduct problems, and learning problems on ADHD diagnosis and treatment differ by sex?

Using the A-TAC continuous scores, in both males and females, symptom severity with respect to inattention and hyperactivity/impulsivity increased the likelihood of receiving a clinical ADHD diagnosis (Table 3) (for example, for males, with each unit increase on the inattention scale the odds of having a clinical diagnosis of ADHD increased by 1.67, whereas in females the odds increased by 1.73). Co-occurring conduct and learning problems were also associated with an increased likelihood of ADHD diagnosis in both males and females. Odds ratios were higher across all predictors in females than males, although these differences were small and non-significant for inattention and learning problems. Interaction analyses revealed sex-by-symptom interactions for hyperactivity/impulsivity (OR 1.08, 95% CI 1.01, 1.15, *p *= 0.03) and conduct problems (OR 1.43, 95% CI 1.09, 1.87, *p *= 0.01), suggesting that externalising symptoms of hyperactivity/impulsivity and conduct problems are more strongly associated with the prediction of clinically diagnosed ADHD in females than in males.

**Table 3:** Predictive value of core ADHD symptoms and co-occurring problems on clinical ADHD diagnosis (based on the National Patient Register) in males and females

Characteristic^a^	Males		Females		Interaction (sex-by-symptom)	
	OR (95% CI)	*P*	OR (95% CI)	*P*	OR (95% CI)	*P*
Inattention	**1.67 (1.61, 1.74)**	**<.001**	**1.73 (1.63, 1.84)**	**<.001**	1.02 (0.96, 1.10)	.43
Hyperactivity/impulsivity	**1.55 (1.49, 1.61)**	**<.001**	**1.68 (1.58, 1.77)**	**<.001**	**1.08 (1.01, 1.15)**	**.03**
Conduct problems	**2.79 (2.41, 3.25)**	**<.001**	**4.09 (3.20, 5.23)**	**<.001**	**1.43 (1.09, 1.87)**	**.01**
Learning problems	**2.53 (2.28, 2.81)**	**<.001**	**2.87 (2.45, 3.35)**	**<.001**	1.11 (0.93, 1.33)	.25

Bold data signify statistical significance of the tests

All models were adjusted for familial clustering, year of birth, and SES

*OR* Odds Ratio (95% confidence interval)

^a^Data were missing on some variables; all available data were used in analysis

Symptom severity with respect to hyperactivity/impulsivity increased the likelihood of being prescribed ADHD medication in both sexes (Table 4). Inattention and conduct problems were associated with an increased likelihood of being prescribed medication in females, but not in males. Interaction analyses revealed sex-by-symptom interactions for hyperactivity/impulsivity (OR 1.24, 95% CI 1.02, 1.50, *p *= 0.03) and conduct problems (OR 2.20, 95% CI 1.05, 4.63, *p *= 0.04), suggesting that externalising symptoms of hyperactivity/impulsivity and conduct problems influence being prescribed pharmacological treatment for ADHD to a greater extent in females than males. Learning problems did not predict medication treatment in the sample overall, or for either males or females (Table 4).

**Table 4:** Influence of core ADHD symptoms and co-occurring problems on prescription of ADHD medication (in the Prescribed Drug Register) in males and females with a clinical diagnosis of ADHD (in the National Patient Register)

Characteristic^a^	Males		Females		Interaction (sex-by-symptom)	
	OR (95% CI)	*P*	OR (95% CI)	*P*	OR (95% CI)	*P*
Inattention	1.08 (.98, 1.20)	.12	**1.22 (1.05, 1.42)**	**.01**	1.13 (0.95, 1.34)	.16
Hyperactivity/impulsivity	**1.12 (1.02, 1.23)**	**.01**	**1.37 (1.15, 1.64)**	**.001**	**1.24 (1.02, 1.50)**	**.03**
Conduct problems	1.04 (.78, 1.36)	.76	**2.29 (1.09, 4.82)**	**.03**	**2.20 (1.05, 4.63)**	**.04**
Learning problems	1.05 (.79, 1.39)	.75	0.83 (.56, 1.23)	.36	0.84 (0.53, 1.31)	.44

Bold data signify statistical significance of the tests

All models were adjusted for familial clustering, year of birth, and SES

*OR* Odds Ratio (95% confidence interval)

^a^ Data were missing on some variables; all available data were used in analysis

For mean scores of clinically diagnosed males and females stratified by medication prescription, see Supplementary Table 2

## Discussion

This large population-based study investigated the role of sex differences in ADHD symptoms and co-occurring conduct and learning problems on clinical diagnosis and pharmacological treatment of ADHD. The main finding was that the predictive association of hyperactive/impulsive and conduct symptoms on ADHD diagnosis and treatment status was stronger in females than in males. We also found, consistent with previous findings, greater ADHD symptoms and co-occurring conduct and learning problems in males than females at the population level [2, 14], more males than females received a clinical diagnosis of ADHD, and that clinically diagnosed males and females showed similar symptom severity [5, 15, 24, 25], except for higher inattention scores in males (which is not in line with the most recent meta-analysis showing greater inattention in females, but the effect size was modest: *d *= 0.21).

Severity of all the symptom domains assessed increased the likelihood of having a clinical diagnosis of ADHD in both males and females. Across all domains, odds ratios were slightly higher for females, suggesting greater deviation from their typical behaviour, and may indicate a greater symptom threshold requirement for referral and diagnosis in females. Significant sex differences were found for the predictive value of hyperactivity/impulsivity and conduct problems, with these externalising behaviours being stronger predictors of diagnosis in females than males. This finding is consistent with a previous study showing that girls with externalising symptoms were referred at a younger age than boys with similar behavioural problems [24]. One explanation for this finding is that externalising symptoms are in greater contrast to what is perceived as normative behaviour in females [7, 24, 26]. Indeed, we found lower levels of hyperactivity/impulsivity and conduct problems in females versus males without a diagnosis of ADHD (i.e., lower baseline levels). These results may also suggest that externalising behaviours drive referral for ADHD [7], speaking to the view that externalising behaviours are more likely to get females clinical recognition for their symptoms. Finally, our finding that externalising behaviours are stronger predictors of diagnosis in females than males suggests that females with ADHD may be more easily missed in the ADHD diagnostic process unless they have prominent externalising problems. This may suggest that the current diagnostic criteria and/or clinical practice are somewhat biased towards the male presentation of ADHD, and has implications for clinical training related to sex role socialisation.

Both hyperactivity/impulsivity and conduct problems were also stronger predictors of ADHD medication status in females compared to males, despite clinically diagnosed males and females being equally as likely to be prescribed ADHD medication. This suggests that if females display less prominent externalising behavioural problems they are less likely to be prescribed medication, whereas males may be prescribed medication based on ADHD diagnostic status alone.

We found no difference in learning problem scores by sex in those with ADHD, which is in contrast to previous research showing more pronounced learning and school-related problems in males with ADHD than in females [14, 15]. Furthermore, the predictive association between learning problems and ADHD diagnosis was similar in males and females. However, learning problems were not associated with being prescribed medication, suggesting that learning problems may not be pertinent to pharmacological treatment decisions for children with ADHD, and in cases where learning problems are particularly prominent in the presentation, alternative interventions may be adopted. The relevance of learning problems to referral, diagnosis, and treatment of ADHD may also differ across countries, where differing importance may be placed on these difficulties in the diagnostic process. It would be interesting to see if findings regarding the impact of learning problems on diagnosis and treatment are replicated in other countries.

Our study found that the combined presentation was the most common ADHD presentation in children with a clinical diagnosis. In contrast, among the children meeting ADHD criteria based on parent-rated symptoms, the inattentive presentation was the most common, which is consistent with some previous research [3, 7, 14, 27, 28], but not all studies [1, 29]. This suggests that some children with primarily inattentive symptoms may not get diagnosed with ADHD. It is possible that: (1) children with predominantly inattentive symptoms are referred but may receive alternative diagnoses in the absence of externalising behaviours [7, 11]; (2) children with the inattentive presentation may be perceived as less impaired and their behaviour as less problematic in comparison to disruptive behavioural problems [30, 31]. These possibilities may be of particular relevance to females, since we found a greater percentage of females than the percentage of males presented with predominately inattentive symptoms at the population level. This could partially explain the greater number of males than females in clinical samples of children with ADHD compared to non-referred samples [14].

This study represents one of the largest samples used to investigate sex differences in ADHD. In addition, previous studies have investigated sex differences in either clinical *or* population samples; here, we uniquely bring the two together. This enabled investigation of a population-based and clinical sample for which there is not an ascertainment bias, overcoming limitations of studies using one type of sample alone. Prevalence rates in this cohort are in line with expectations and suggest an overall reasonable detection of ADHD in Sweden. The administrative prevalence of ADHD was 3.28%, and the symptomatic prevalence based on the A-TAC was substantially higher at 12.9% as impairment criteria and symptom pervasiveness across settings were not applied, consistent with previous estimates of ADHD classification based on symptom counts alone [3]. However, this rate is similar to estimates from other community studies that apply impairment criteria [2]. Of note, another potential explanation for the prevalence difference between clinically diagnosed ADHD and symptomatic ADHD is that males and females who have less pronounced levels of externalising behaviours and a predominantly inattentive presentation are less often clinically diagnosed. Among children clinically diagnosed with ADHD, the ratio of males to females was 2.5:1, which is somewhat lower than previously reported [5, 9]. Of the entire CATSS sample, the ratio of males to females meeting symptomatic threshold was 1.8:1, which is consistent with previous findings [3]. Thus, the difference in ratios of males to females in the clinical and population sample was small, and findings of this study may not generalise to countries with lower (or higher) administrative prevalence rates.

Our findings should be considered in the context of some limitations. Our findings are telling us about diagnosis patterns and do not provide information about referral patterns. It is possible that a number of children are referred but receive alternative diagnoses or are not considered sufficiently impaired by symptoms to obtain a diagnosis. Future studies should investigate such hypotheses. We were also unable to confirm whether additional children from CATSS should have a clinical diagnosis of ADHD (i.e., children who are potentially ‘missed’ in the community); as an epidemiologic cohort, our study did not have objective clinician or research interviews. Furthermore, we relied on parent-ratings of ADHD symptoms and co-occurring problems, which may be influenced by sex-specific biases and expectations [26, 32]. For example, there is some evidence that parents may underrate females’ ADHD symptoms compared to males [32]. A further limitation is that, unfortunately, our main analyses did not examine co-occurring internalising problems due to a reduced number of CATSS participants completing these measures. Sex differences in internalising problems have been reported [9] and further research is needed to explore the predictive associations with diagnosis and treatment of ADHD. We were also unable to explore potential sex differences in referral to non-pharmacological interventions for ADHD. Finally, the study was carried out in a twin sample and findings may not generalise to singletons; for example, twins are more likely to have lower birth weight compared to singletons which is a risk factor for ADHD [33, 34]. Findings require replication in a non-twin sample.

These limitations notwithstanding, overall the current findings highlight the importance of the clinical presentation of ADHD as it can influence diagnosis and treatment decisions differentially in males and females, and the prominence of different symptoms clearly matters. Externalising behavioural problems were more predictive of diagnosis and pharmacological treatment in females than males, perhaps because they contrast more with perceptions of normative behaviour in females. One interpretation of these findings is that females with ADHD may be under-identified in the absence of prominent externalising problems.

We hope that our findings encourage more research in this area to foster greater understanding of sex-specific diagnostic patterns and more effective recognition, diagnosis, and treatment of ADHD in females in clinical, educational, and other settings.

### Disclosures

Paul Lichtenstein has served as a speaker for Medice. King’s College London received funds for consultancy and other work by Philip Asherson, which has been used to support departmental research on ADHD: consultancy to Shire, Eli-Lilly, and Novartis, regarding the diagnosis and treatment of ADHD; educational/research awards from Shire, Eli-Lilly, Novartis, Vifor Pharma, GW Pharma, and QbTech; speaker at sponsored events for Shire, Eli-Lilly, and Novartis—all outside the submitted work. Henrik Larsson has served as a speaker for Eli-Lilly and Shire and has received a research grant from Shire; all outside the submitted work. Florence Mowlem, Mina Rosenqvist, and Joanna Martin report no potential conflicts of interest.

## Electronic supplementary material

Below is the link to the electronic supplementary material.

Supplementary material 1 (DOCX 17 kb)
