# Supplementary material for: Sex differences in predicting ADHD clinical diagnosis and pharmacological treatment
Source: Eur Child Adolesc Psychiatry. 2018 Aug 10;28(4):481–9. doi: 10.1007/s00787-018-1211-3 (PMC6445815; doi:10.1007/s00787-018-1211-3)
Supplement: Supplementary file 1 — Supplementary material 1 (DOCX 17 kb) [file 787_2018_1211_MOESM1_ESM.docx]

**Supplementary Online Content**

Mowlem, F.D., Rosenqvist, M.A., Martin, J., Lichtenstein, P., Asherson, P., & Larsson, H. (2018). Sex differences in predicting ADHD clinical diagnosis and pharmacological treatment. *European Child & Adolescent Psychiatry.*

**Supplementary Table 1.** Characteristics of males and females in the entire sample (means and SD unless otherwise stated)

**Supplementary Table 2.** Characteristics of clinically diagnosed males and females stratified by medication prescription (based on the Prescribed Drug Register) (means and SD)

**Supplementary Table 1.** Characteristics of males and females in the entire sample (means and SD unless otherwise stated)

| **Characteristic ^a^** | **Overall**  **(n=19,804)** | **Males**  **(n=10,029)** | **Females**  **(n=9,775)** | ***p*** | **Cohen’s *d*** |
| --- | --- | --- | --- | --- | --- |
| Total ADHD | 2.06 (3.13) | **2.49 (3.46)** | **1.62 (2.68)** | **< .001** | 0.28 |
| Inattention | 1.05 (1.75) | **1.29 (1.92)** | **0.81 (1.52)** | **< .001** | 0.28 |
| Hyperactivity/Impulsivity | 1.01 (1.71) | **1.20 (1.88)** | **0.81 (1.48)** | **< .001** | 0.23 |
| Conduct | 0.10 (0.37) | **0.12 (0.41)** | **0.08 (0.32)** | **< .001** | 0.11 |
| Learning | 0.27 (0.61) | **0.30 (0.63)** | **0.25 (0.58)** | **< .001** | 0.08 |

Bold data signify statistical significance of the tests

All models were adjusted for familial clustering, year of birth, and SES

^a^ Data were missing on some variables; all available data were used in analysis

**Supplementary Table 2**. Characteristics of clinically diagnosed males and females stratified by medication prescription (based on the Prescribed Drug Register) (means and SD)

|  | **Males** | | **Females** | |
| --- | --- | --- | --- | --- |
| **Characteristic ^a^** | **Prescribed medication**  **(n=396)** | **Not prescribed medication**  **(n=70)** | **Prescribed medication**  **(n=156)** | **Not prescribed medication**  **(n=28)** |
| Inattention | 4.67 (2.82) | 4.25 (2.72) | 4.23 (2.97) | 2.79 (2.67) |
| Hyperactivity/Impulsivity | 4.25 (3.20) | 3.41 (2.88) | 4.10 (3.23) | 1.88 (2.03) |
| Conduct problems | 0.58 (0.99) | 0.58 (0.90) | 0.70 (1.08) | 0.25 (0.48) |
| Learning problems | 0.98 (1.01) | 0.96 (1.08) | 1.07 (1.12) | 1.30 (1.15) |

^a^ Data were missing on some variables; all available data were used
